# Enablers and Constraints of STEM Programme Implementation: an External Change Agent Perspective from a National STEM Programme in Finland

**DOI:** 10.1007/s10763-022-10271-9

**Published:** 2022-04-14

**Authors:** Tiina Mäkelä, Ari Tuhkala, Matias Mäki-Kuutti, Juhani Rautopuro

**Affiliations:** Finnish Institute for Educational Research, University of Jyväskylä, Jyväskylä, Finland

**Keywords:** Constraints, Effectiveness, Enablers, Programme, STEM

## Abstract

An international need exists for effective programmes that will enhance learners’ interest in studies and careers related to science, technology, engineering and mathematics, i.e. STEM. When considering the impact of STEM programmes, it is important to identify what can enable or constrain effective programme implementation. As such, enablers can be systematically supported and constraints tackled to maximise a programme’s impact. This study considers a nationwide STEM programme in Finland that involved 450 teachers and their learning communities. The study followed an interpretative paradigm, collecting data through semi-structured interviews and analysed with data-driven thematic analysis. The study participants were 14 external change agents operating between participating learning communities and the national programme administration and, thus, had a valuable overview on how the programme evolved. The most important enablers of effective programme implementation were versatile support, programme flexibility and long-term vision. Two major constraints were limited resources and collaboration challenges. Considering the findings of this study can help design effective STEM programmes by providing various support mechanisms for educators, particularly scaffolding; peer and school administrator support; flexibility to enable embedding activities and adapting programme objectives to participants’ everyday work; long-term vision to support both lifelong learning and a continuum between different STEM programmes; sufficient time and economic resources to achieve a long-term impact; and collaboration and networking opportunities at the local, regional, national and international levels.

## Introduction

The results of international assessment programmes, such as the Programme for International Students Assessment (PISA) and Trends in International Mathematics and Science Study (TIMSS), indicate that an urgent need exists to enhance learners’ interest in mathematics and science (Mullis et al., 2020; Organisation for Economic Cooperation and Development [OECD], 2016). The number of students enrolling and remaining in studies related to science, technology, engineering and mathematics (STEM) has been found to be low internationally, and many choose to pursue other disciplines after showing an initial talent and interest in STEM (van den Hurk et al., 2019). Consequently, the lack of interest in STEM careers has already caused difficulties in recruiting professionals to fill open STEM positions (DeCoito, 2016; Martín-Páez et al., 2019).

One possible way to tackle this issue is to implement effective STEM programmes. For example, previous STEM programmes have tried to increase learners’ interest in STEM subjects and careers, decrease dropout rates in STEM studies and increase female or minority group participation (van den Hurk et al., 2019). Effective STEM programmes have been found to require strong administrative support and professional development opportunities for educators (Icel, 2018). The quality of STEM afterschool programmes (Allen et al., 2019) and parental involvement (Dou et al., 2019) has also been found to increase STEM engagement and career interest. More research is, however, needed to better identify the characteristics of effective STEM programmes (DeCoito, 2016; van den Hurk et al., 2019), which is also reflected in the increasing number of studies focusing particularly on policy, curriculum, evaluation and assessment in STEM (Li et al., 2020).

The effectiveness of educational programmes is usually evaluated by comparing whether the actual impact is the same as the intended impact (Kreber & Brook, 2010). However, in addition to impact evaluation per se, it is crucial to identify the factors that may enable or constrain effective programme implementation (Wilson, 2020). To answer this call, the research objective of this study is to identify enablers (facilitators, good practices) and constraints (barriers, hindrances, impediments) that affect effective STEM programme implementation.

This study considers the “LUMA2020” STEM programme, a large-scale national programme in Finland that involved 160 learning communities with 450 teachers and their learner groups from early childhood, basic education and upper secondary education. According to the official programme impact evaluation report, 87% of the respondents (total *n* = 160) agreed that the programme supported the development of formal STEM education, 90% agreed it supported participants’ professional development, 92% agreed it increased their own interest towards STEM, and 93% agreed it increased the learner group members’ interest towards STEM (Aksela & Kiviluoto, 2020). Although the participants of the programme seemed very satisfied and the programme objectives were fulfilled, we wanted to better understand the factors that may have enabled and constrained the programme’s effective implementation process. For this purpose, we designed a study with an interpretative approach: interviewing *external change agents* of the programme and conducting a data-driven thematic analysis. In the next section, we discuss the idea of external change agents in more detail and argue why they were such critical subjects to study. This is followed by a description of the focus of this study, the LUMA2020 programme.

### External Change Agents’ Perspective

*Change agents* can be described as change generators, taking the lead in the change process (Ottaway, 1983). Important actions of change agents include planning, bringing ideas, problem-solving, obtaining resources, providing support and facilitating participatory decision-making as well as training and workshops (Miles et al., 1988). Change agents can be understood as leaders, driving school reforms at the school, community, district and state levels, ideally in the form of joint collaboration (Fullan, 2007).

*Internal change agents* initiate change processes within learning communities. They can be, for example, school directors or expert teachers who are willing to renew or develop existing practices. In comparison, external change agents come from outside the learning community; thus, they often have more diverse experiences in change processes as they have been working with various schools (Tajik, 2008).

*External change agents* can take the roles of facilitators, critical friends or technical experts (Tajik, 2008). As facilitators, they assist learning communities in identifying resources, expertise and key stakeholders, and they provide support in implementation processes and stimulate educators’ motivation to change. As critical friends and technical supporters, they collaborate with educators in solving their challenges and support them in enhancing their knowledge, skills and teaching methods, as well as provide expert ideas to address the challenges of implementing an innovation. Consequently, external change agents are relevant in supporting diffusion, training and community development, team learning, self-reflection and leadership (Tajik, 2008).

**Fig. 1:**
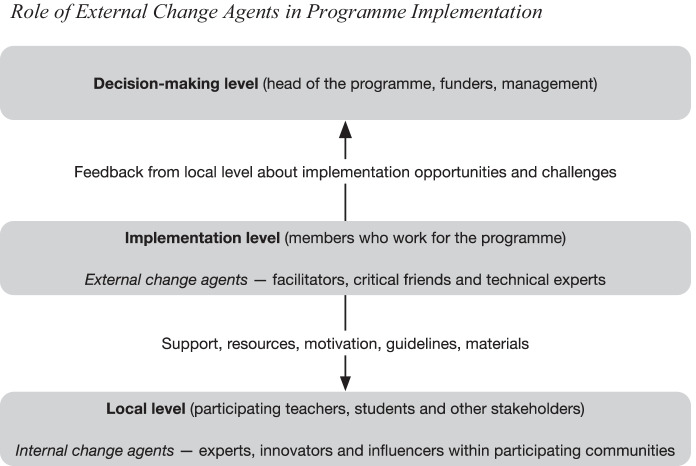
Role of external change agents in programme implementation

Figure 1 describes how the role of external change agents is to provide support, resources and guidelines for the local learning communities and motivate them to fulfil the programme objectives. Moreover, external change agents listen to emerging local needs and impart valuable knowledge about implementation opportunities and challenges at the decision-making level. As such, we argue that they are in the best position to identify enablers and constraints of effective programme implementation, not only because of their involvement in several different learning communities, but also because they operate between participant learning communities and decision-makers. In other words, they serve as mediators between the local grassroots level and the official decision-making level.

### LUMA2020 Programme Description

The LUMA2020 programme was initiated by the Finnish Ministry of Education and Culture and executed by the LUMA Centre Finland, a network organisation of 13 regional LUMA Centres located in 11 universities throughout the country. LUMA is an abbreviation of the Finnish words for science (luonnontiede) and mathematics (matematiikka), but it is commonly used to cover technology and engineering education as well, i.e. STEM. The programme aimed to support children’s and youth’s inspiration and learning motivation regarding STEM and increase the quality of STEM teaching and learning for all ages. The programme’s main goals were to (1) develop STEM-related formal education from early childhood to secondary education, (2) improve teachers’ and educators’ professional development related to STEM subjects and (3) advance non-formal and out-of-school STEM activities (see also Aksela, 2019).

The programme consisted of four themes: sustainable development, e.g. climate change; math around us, e.g. math and art; technology around us, e.g. artificial intelligence; and my LUMA, a theme integrating various subjects. In all theme groups, the aim was to create multidisciplinary learning modules required by the Finnish National Core curricula (Finnish National Agency for Education, 2014). As is typical in STEM education (Martín-Páez et al., 2019), particular emphasis was placed on inquiry- and project-based learning, as well as integrating various subjects related to science, technology, engineering and mathematics as well as other subjects to solve real-world problems.

**Fig. 2:**
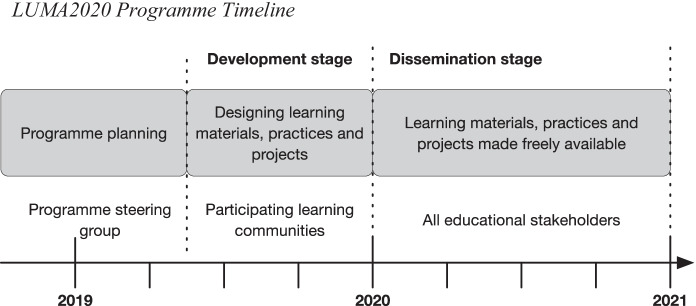
LUMA2020 programme timeline

The programme timeline is depicted in Fig. 2. The programme planning involved applying for funding, defining project goals, recruiting learning communities and assigning theme team members by the programme steering group. The development stage started in May 2019, where the participating learning communities designed educational materials, practices and science projects for the learning modules. During this stage, the regional LUMA centres provided the learning communities with research-based knowhow, practices, models and materials, as well as support, networks and in-service training related to the creation of the learning modules. In the dissemination stage, the developed materials, practices and projects were made available to all Finnish educational stakeholders via open online courses and materials. The programme outcomes were presented to the public on national LUMA days 3–5 June 2020, although the workshops and lectures were organised virtually because of the pandemic. On 11 December 2020, a final national online seminar took place, presenting the programme outcomes.

**Fig. 3:**
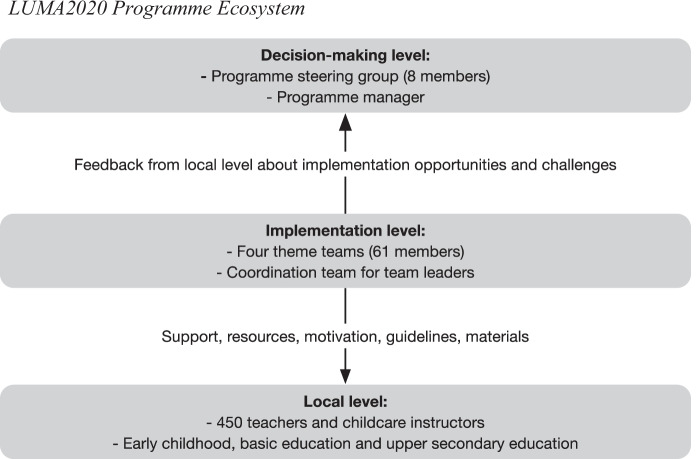
LUMA2020 programme ecosystem

Figure 3 presents the LUMA2020 programme ecosystem. The main administrative responsibility was held by the steering group, which involved eight educational experts: two representatives from the Ministry of Education and Culture, the head of the LUMA Centre Finland and five professors from partner universities. A programme manager was hired to coordinate the actions at the implementation level. The implementation level consisted of four teams, one for each programme theme. Altogether, 61 persons from regional LUMA Centres worked in the four theme teams. Every team had two leaders, who also operated in the national coordination team. The coordination team communicated with the programme manager, who reported the actions to the steering group. They also coordinated the support provided to 450 educators, who were developing their projects on a local level.

## Method

### Participants

First, we assessed the LUMA2020 programme operators to identify the external change agents for the study (Fig. 2). The members of the steering group and the programme manager represent the decision-making level; thus, they were not involved in implementing the programme. Instead, it was the responsibility of the four theme teams to distribute and disseminate the support to the local learning communities to enable them to design the multidisciplinary learning modules.

Hence, it was rather evident that the team leaders (*n* = 8) were the first external change agents who we invited to the interviews. However, we noticed that the team leaders represented only four LUMA centres; therefore, we extended the invitation to three more LUMA centres to achieve a better geographical representation of LUMA centres around Finland, including areas with Swedish- (west coast) and Sami- (Lapland) speaking populations. Participants in these additional interviews were familiar with all theme teams and, thus, had an overall view of the work in different theme teams. All participants also had experience working with other STEM programmes prior to LUMA2020. Finally, we were able to involve interviewees from 7 out of 13 LUMA centres. Ultimately, seven interviews (total *n* = 14, females *n* = 10, males *n* = 4) were organised during March and April 2020, using video conferencing tools (see Table 1). One interview involved three participants, while due to a last-minute cancellation from one participant, one individual interview was conducted. We had decided to interview a minimum of two individuals simultaneously to facilitate a dialogue, thereby promoting more reflection and varying perspectives. The individual interview, however, was also considered to enrich the data otherwise collected in group interviews.

**Table 1:** Participants per theme teams

Theme teams/interviews	Math around us	Technology around us	My LUMA	Sustainable development
Interview 1	*n* = 2			
Interview 2		*n* = 2		
Interview 3			*n* = 2	
Interview 4				*n* = 2
Interview 5	*n* = 3			
Interview 6	*n* = 2			
Interview 7	*n* = 1			

### Data Collection

We found semi-structured interviews to be the most adequate method to pursue our research objective. We designed the interview structure in collaboration, but the interviews were executed by the first author. The first interview questions, which focused on the programme objectives, were designed to support collecting qualitative data on whether the actual impact was perceived to be the same as the intended/expected impact and whether the actual impact was considered desirable (see Chalmers & Gardiner, 2015; Kreber & Brook, 2010). The beginning of the interview thus focused on how well the interviewees thought the programme’s main objectives had been accomplished, that is, (1) how the LUMA2020 programme supported the development of children’s and young people’s formal education from early childhood to secondary school levels; (2) how LUMA2020 supported the development of teachers’ and educators’ competencies at various educational levels; and (3) how LUMA2020 supported the development of children’s, young people’s and families’ non-formal STEM education activities. There was also a question related to (4) how LUMA2020 supported the creation of local, regional, national and international networks. This was related to the LUMA Centre Finland organisation’s overall objectives related to network creation. Finally, there were specific questions related to (5) good practices and applicable models that were applied during the programme and (6) challenges that obstructed the programme objectives. These questions were directly related to research questions of the specific sub-study described in this article (see also Pinkerlman et al., 2015; Wilson, 2020). The final question was oriented towards (7) ways to develop similar actions in the future.

The interview structure was sent to the interviewees before the interviews. Participants were instructed to think of questions as representatives of theme teams, thereby acting as experts or subject specialists (Tajik, 2008). It was explained to them that the data would be anonymised; thus, personal details would not be shared outside the research team. Participants were encouraged to express their views in a transparent manner and to critically evaluate the achievement of the programme objectives. When conducting the interviews, with only a few exceptions, all Finnish educational institutions were using distance education due to the COVID-19 pandemic. The interviewer asked, however, that, although this would have an impact on the programme implementation, it would be important to focus on everything that had been achieved before school closure. Interviews lasting from 1.5 to 2 h were recorded and then manually transcribed for analysis.

### Data Analysis

The data were analysed following the data-driven thematic analysis (Braun & Clarke, 2006; Vaismoradi et al., 2016). With data-driven analysis, we refer to the inductive approach of qualitative content analysis, as explained in Elo and Kyngäs (2008). In a deductive approach, the basis of analysis is on an existing theoretical framework that, for example, defines the coding categories that the data fit. An inductive approach, in turn, uses the research question as a starting point, and the data are examined with open coding. As such, the purpose of coding was to identify the enablers and constraints of STEM programmes and then refine the initial codings into higher-level themes that answer the research question. While theoretical models were not used in the data analysis to guide the interpretation, it is acknowledged that the approach is not strictly inductive, because the research questions necessarily resonate with existing theoretical knowledge on the topic.

Within the scope of this article, we focus on interview excerpts that pertain to the good practices and applicable models that were applied during the programme (question 5), as well as challenges that obstructed the programme objectives (question 6), but we also examine other interview excerpts to identify any positive or negative comments that could be related to the research question. The first author read through the transcripts and made some initial remarks. The focus was on significant, i.e. meaningful or relevant, themes from the point of view of the research question, which were commonly but not necessarily repeated in the data. After the open coding stage, initial themes were identified. The reliability of the analysis was strengthened by reviewing the initial themes and the excerpts that contained them in collaboration with all authors. As such, we developed the initial themes by dividing or combining them, if necessary, and we organised similar themes together under higher-level groups until a consensus was reached on the final structure. In the final theme structure, for instance, the subtheme “pre-service and in-service teacher collaboration” was merged with the subtheme “peer support”, and “educators’ busy schedules” was merged with the more generic subtheme “time for planning and implementation” to avoid an overly extensive list of subthemes (Table 2).

**Table 2:** Enablers of and constraints on effective STEM programme implementation

Enablers of effective STEM programme implementation	Constraints on effective STEM programme implementation
**Versatile support** In-service training and workshops Materials and equipment Scaffolding Peer support Support from school administration Extensive networks **Programme flexibility** Visits to learning communities Embedding activities to everyday work Online communication Flexible programme objectives **Long-term vision** Continuum from early years to adult education Parental involvement Continuum between different tasks and projects Evaluating programme effectiveness	**Limited resources** Time for planning and implementation Lack of financial resources Balancing between tasks Resources for continuity and long-term impact **Collaboration challenges** Involving external stakeholders Regional and national collaboration Language barriers Reaching out towards less-active communities

Citations from interviewees were selected and translated into English for the purpose of this article and to present excerpts that represent external change agents’ direct voices. The translations aim to maintain the conversational style and expressions used by the interviewees, but filler words were left out of the excerpts to clarify the message. The citations are marked as interviews from 1 to 7, e.g. Interview 1 and Interview 2, and participants are marked from a to c, e.g. Interview 1a.

## Results

Based on the data-driven thematic analysis, the enablers and constraints were organised into five categories from four to six subthemes (Table 2). The main enabler categories were versatile support, programme flexibility and long-term vision. Constraint categories were limited resources and collaboration challenges. The following subheadings present each category and their subthemes.

### Versatile Support

#### In-Service Training and Workshops

The LUMA centres offered in-service training and workshops related to programme themes and approaches, particularly to project-based learning. Educators were also offered example sessions with their learner groups, which they could then use in their own teaching. These sessions were offered by both visiting participating educational organisations and receiving groups at regional LUMA centres. For example:

Teachers from one lower secondary school visited us with their student groups, and we provided them with example sessions related to their projects combining maths, physical education and medical sciences. (Interview 1a)

#### Materials and Equipment

The LUMA centres provided many online resources, such as lesson plans and video demonstrations. Interviewees reported that, instead of merely providing the materials, it was important to actively present them and support educators by recommending materials that responded to each educator’s personal development needs and the project they were developing. Regional LUMA centres either have their own laboratories and equipment or share facilities with university departments representing the natural sciences or teacher education. As a part of the LUMA2020 programme, educators could either borrow laboratory equipment from LUMA centres or visit LUMA labs with their learner groups and perform some experiments while being supported by the representatives of LUMA centres:

Teachers and their groups came to do some experiments at the teacher training laboratories at our university, where they could also familiarise themselves with authentic research equipment and research. (Interview 5c)

#### Scaffolding

Representatives of regional LUMA centres offered personalised and timely long-term support, i.e. scaffolding, to the educators for planning and implementing their projects. They also collaborated with the educators’ in designing learning modules for transversal, project-based and active learning. Interviewees viewed this form of support as more efficient than traditional in-service training:

The deepest way to impact teaching is by working hand-in-hand with teachers in their everyday work. (Interview 7a)

#### Peer Support

Mutual learning opportunities between participant educators were facilitated via regular local face-to-face and online meetings and creating forums, e.g. social media, for sharing experiences and ideas. This form of support was seen as more effective than top-down support. Meetings with the educators were described as having a relaxed and confidential atmosphere. Peer support was also provided between in-service and pre-service teachers. As a part of the LUMA2020 programme, pre-service teachers were sent to participating learning communities to support teachers in their project work, providing mutual learning experiences between teachers and students:

A very close group of early childhood educators were producing materials together and keeping in touch, and they will also likely stay in touch when the programme finishes. (Interview 5a)

#### Support from School Administration

It was seen as important that school directors supported educators and were committed to programme implementation. Directors were expected to ensure that substitute teachers would be on hand to cover the time educators would be spending outside of school in meetings and trainings. At best, they themselves participated in the programme:

When enrolling in the programme, the requirement was that it had been agreed with the director that teachers could use their time with the programme. (Interview 3a)

#### Extensive Networks

Interviewees viewed various local, regional and national networks that were used and strengthened during the programme as important enablers of quality programme implementation. Networks were seen as important for disseminating and transferring good practices. Existing networks were harnessed, and new connections were created. The programme succeeded in involving learning communities representing all educational levels, including some vocational institutions. In addition to 11 universities that have LUMA centres, there were collaborations with universities of applied sciences. The programme enabled and strengthened collaboration between representatives of various LUMA centres. National collaboration and the sharing of information took place both in face-to-face and online meetings. The LUMA centres also facilitated international collaboration by translating some materials into English, Swedish and Sami:

It has been nice to hear what has been done in other centres. You learn from these practices. (Interview 2b)

### Programme Flexibility

#### Visits to Learning Communities

Representatives of LUMA centres visited learning communities instead of requiring educators to travel. This required less time and monetary resources on the part of the learning communities. This was particularly important for learning communities located far away from their regional LUMA centres:

As there are long distances from one place to another in some regions, representatives of LUMA centres have visited schools and done action-based workshops there. (Interview 4a)

#### Embedding Activities in Everyday Work

It was considered important to ensure that the activities would be integrated into educators’ everyday work or professional development activities. In this way, participation in the programme would not appear to be an additional burden but an opportunity to obtain support for work in one’s own community:

One of the strengths (of the programme) is its concreteness, that everything can happen at one’s own unit, working among children, and not outside one’s work. (Interview 6a)

#### Online Communication

Online meetings both between representatives of LUMA centres and with the programme participants were organised as a less resource-consuming activity for all. Online communication was seen as important particularly in areas with long distances, such as Lapland. In addition, the COVID-19 pandemic forced the need to replace face-to-face activities with online alternatives. This was seen as positive because it catalysed the creation of online options, which were deemed useful in normal situations as well. In addition to formal learning, non-formal science camps and clubs were organised completely or partially online:

We created virtual workshops with video instructions for experiments that children can do remotely. Teachers can choose and recommend them to their group. (Interview 7a)

#### Flexible Programme Objectives

Flexibility was built into the programme objectives for each participant. They could, for instance, decide whether they wanted to only plan or also implement the project during the programme:

Some participants decided that they would focus on planning and implementing their projects only later on, and that was okay too. (Interview 4a)

### Long-Term Vision

#### Continuum from Early Years to Adult Education

Interviewees deemed it important to put effort into early childhood education and the first years of primary school. Inhibiting the development of negative perceptions and gaps in achievement from an early age were considered important when aiming to impact children’s and youth’s interest in STEM studies and careers in the long-term. In addition to focusing on the work at an early stage, interviewees felt that a long-term impact can be achieved by ensuring continuity in STEM education from early childhood to adult education and connecting STEM teaching and learning at various educational levels. This was already fostered during the programme by supporting networks between learning communities representing different educational levels:

In early childhood education, children can develop their relationship with nature by going to a forest and seeing how there are many different plants. Then, it is possible to deepen that in primary school, based on the development level . . . I see it as a continuum. (Interview 4b*)*

#### Parental Involvement

The programme also aimed to involve parents and other family members, such as grandparents, in activities. Family members were considered important influencers of children’s and young people’s future career choices. Interviewees viewed collaboration with families, particularly in early childhood education, as natural and frequent. For example:

There was a science café at one daycare centre once a month at the end of the day in which families could participate. There were different themes, science tasks, the observation of the weather and the environment, statistics about the need to wear mud pants and so on . . . (Interview 4b)

#### Continuum Between Various Tasks and Projects

Also deemed important was that the different tasks and projects/programmes in which the representatives of the LUMA centres participate are connected and form a meaningful whole. Activities developed in one programme could be further developed in future programmes, and future actions could be constructed based on the earlier actions:

It’s important to think about how to build on what has already been developed and how to improve existing materials. (Interview 1b)

#### Evaluating Programme Effectiveness

Evaluating programme effectiveness using both quantitative and qualitative methods was seen as beneficial in terms of developing the work and future programmes offered by LUMA centres:

The evaluation is one of the programme strengths from the beginning to the end, a professional and research-based approach to effectiveness evaluation. (Interview 2a)

### Limited Resources

#### Time for Programme Planning and Implementation

Interviewees commented that there were numerous objectives given the short project lifetime (less than 1.5 years). It was challenging to move quickly from planning to implementation and from implementation to sharing and disseminating the results. The interviewees felt that more time would have been required to concretise the programme objectives and plan the actions, both between representatives of different LUMA centres and between LUMA centres and learning communities. It was also reported that local differences should be considered when planning actions at the national level. Educators were also seen as very busy in their everyday work. Upper secondary school teachers were considered particularly busy due to curricular reform and the digitalisation of the matriculation examination at that level. Also raised was that when the programme started, many learning communities had already planned their school year, and it was challenging to add new activities. Additionally, differences between the autumn and winter holidays in various parts of Finland made it difficult to create a functional schedule for activities at the national level:

Teachers have been very busy. They have not had much time for anything. We have tried to tell them that they do not have to do anything additional, but this is just to support their everyday work. (Interview 1a).

#### Lack of Financial Resources

Due to a lack of financial resources to recruit more personnel in smaller LUMA centres, the same person had to find time to oversee all four programme themes. In some cases, educators did not have the technologies needed for activities, or group sizes were too large for laboratory experiments, particularly in some upper secondary schools. It was reported that learning communities would need additional funding to effectively participate in these types of programmes. It was deemed important that the programme objectives were planned realistically in line with the programme funding and duration and that they focused on making a deep impact on a smaller number of objectives, instead of aiming to achieve various goals with limited resources:

We were concerned that so many different things were supposed to be done in one and a half years . . . school projects, online courses, their dissemination . . . (Interview 1a)

#### Balancing Between Tasks

Interviewees viewed the continuum between various tasks and projects as an important enabler of effective programme implementation. In practice, however, it was perceived as challenging that representatives of LUMA centres often had to divide their time between many tasks and projects. This also made it difficult to distinguish between the programme impact and the general impact of the various actions conducted in LUMA centres. Many interviewees not only worked at the regional LUMA centre but were also working as university teachers:

In addition to general coordination responsibilities in our LUMA centre, I work as a coordinator in another project, and then I’m in the LUMA2020 project. There aren’t always enough working hours for everything. (Interview 1a)

#### Resources for Continuity and Long-Term Impact

One challenge is the question of how to maintain the activities and widened networks after the programme finishes. For instance, it was deemed important that LUMA centres continue providing support in maintaining and sharing projects both within one learning community and between learning communities after the programme finished. Participants reported that it was important to find resources to support the deployment and use of new (online) materials. For instance, instructions on how to use materials would be important, particularly among educators with no previous experience using them. It was mentioned that developing and, ultimately, sharing high-quality materials may require various iterations, something that was not possible in a short programme. Further, longer funding periods were considered important in ensuring a deeper impact:

With longer funding periods, there would be more impact. We have done good things and accomplished a lot, but we would have accomplished much more had there been more time for reflection. (Interview 1a)

### Collaboration Challenges

#### Involving External Stakeholders

It was seen as difficult to involve representatives of STEM companies and organisations in the programme. Contacting external STEM experts and organising their visits to learning communities was also difficult. There was a need, for instance, to further develop “an expert bank” for collecting experts, lecturers and organisations interested in collaborating:

We could get more purely commercial collaborators to join in. There have been less of them. That could be developed. (Interview 3b)

#### Regional and National Collaboration

Despite the co-design approach employed in the LUMA2020 programme and the chance for collaborative programme planning, due to the short timeframe allocated for planning, the interviewees felt that the time to consider opinions and suggestions at the local and regional levels was insufficient. This was seen as particularly important when planning the programme. Interviewees also felt that they generally had more success collaborating with the learning communities at the regional level than at the national level. It was also mentioned that some teachers desired more national collaboration because, on a local level, they already knew one another. Additionally, the difficulties involved in the collaboration at the LUMA Centre Finland network were partly seen as related to the need to find the time and match the schedules between LUMA centres nationally:

Agents at regional LUMA centres should be able to have a stronger influence on what is planned to be done. They have lots of good ideas and practical knowledge and experience about what is needed in the field. (Interview 1a).

#### Language Barriers

Involving Sami-speaking learning communities in national collaboration was seen as challenging, as was collaboration between Finnish- and Swedish-speaking teachers:

In addition to a lack of time, teachers may be shy about contacting teachers they do not know, particularly if there is a language barrier, because they may not be so fluent with the second national language. (Interview 6b)

#### Reaching Out Towards Less-Active Learning Communities

Although the programme helped in reaching out to new learning communities, interviewees felt that resources were still directed to a relatively small number of participants. It was deemed important that resources be allocated to recruiting new participants into programme collaboration. A particular need exists to motivate less-active learning communities and educators to become involved in these kinds of programmes:

Many schools have already been active, and they have done different development work already . . . I personally hope we can provide activities to a wider crowd. (Interview 1a)

## Discussion

The key enablers of the LUMA2020 programme were (1) versatile support, (2) programme flexibility and (3) long-term vision. In contrast, (4) limited resources and (5) collaboration challenges were two crucial constraints.

*Versatile support* for participants was perceived as the first main enabler for effective STEM programme implementation. In the LUMA2020 programme, versatile support consisted of in-service training and workshops, materials and equipment, scaffolding, peer support, support from school administration and extensive networks. When considering future STEM programmes, creating opportunities for capacity building and professional development (Icel, 2018) and facilitating teacher collaboration and peer support (Anagnos et al., 2014; Icel, 2018; Milner-Bolotin, 2018; Tytler et al., 2019) seem to be effective ways to provide support to programme participants. Based on the findings of this study, instead of more traditional training sessions, teachers may benefit more from example sessions for educators with their learner groups during their everyday work. Our findings also confirm that in situ training and teacher development programmes, including communities of practice, mentoring and action learning (Chalmers & Gardiner, 2015), and providing support for project-based learning activities (Hall & Miro, 2016) can be viewed among the most effective support mechanisms for teachers.

It is also important to develop appropriate and appealing materials (Allen et al., 2019). Our findings suggest, however, that instead of merely offering materials and equipment, it is important to offer adequate resources based on educators’ specific development needs and STEM project objectives and to provide instructions on how to use materials. Furthermore, encouraging school administrators to support programme participants (Pinkerlman et al., 2015; Wilson, 2020) and creating wide networks and partnerships with various private, public and government agencies at local, regional, national and international levels are considered vitally important when developing STEM education (Basham et al., 2010; DeCoito, 2016).

*Flexibility* of the LUMA2020 programme was perceived as a second major enabler in programme implementation, which included visits to learning communities, online communication, embedding activities into everyday work and flexible programme objectives. Including visits to learning communities into a STEM programme, in conjunction with online communication, widens access for educators with fewer travel possibilities (Basham et al., 2010). Thus, innovations developed in STEM programmes can be embedded into the everyday work of programme participants, making programme participation an opportunity to obtain support, rather than an additional burden. In addition to flexibility in programme implementation, flexible programme objectives provide teachers with the freedom of choice and more space to implement programme activities that support the local learning communities (Wilson, 2020). However, continuously refining the plans based on different contextual factors demands reaching a balance between establishing shared goals and individual interests (Tajik, 2008).

The third identified enabler in the LUMA2020 programme, *a long-term vision*, meant continuity from the early years to adult education, parental involvement, a continuum between different tasks and projects and evaluating programme effectiveness. Initiating future STEM programmes for early childhood education is a good starting point to support children’s interest towards STEM (Tippett & Milford, 2017), such as in the form of informal STEM-related conversations (Dou et al., 2019). As Roth and Eijck (2010) maintained, life should be considered a minimal unit in lifelong, life-wide and life-deep STEM learning. In this project, lifelong and life-wide STEM learning was facilitated by creating networks between educational levels and between formal and non-formal education.

Likewise, STEM programmes raising awareness about the importance of STEM education among parents, e.g. through parental involvement, are seen as important to prevent dropouts from STEM education, increase interest towards STEM and develop a STEM identity (Dou et al., 2019; Eshach, 2007; Millar et al., 2019; van den Hurk et al., 2019). Furthermore, future STEM programmes should complement previous programmes and actions to avoid becoming disconnected, episodic or fragmented (Tajik, 2008) and put effort into evaluating the programme’s effectiveness to identify future development needs (van den Hurk et al., 2019).

The first main constraint in the LUMA2020 programme was related to *limited resources*: the time for planning and implementation, a lack of financial resources, balancing between tasks and resources for continuity and long-term impact. The LUMA2020 programme objectives were perceived as highly ambitious, and participants felt that there was a lack of resources, compared to the objectives. Thus, reserving sufficient time resources for planning and implementing STEM programmes are necessary (Pinkerlman et al., 2015). Similar to our study, in addition to limited temporal resources, limited resources in terms of funding and staffing and large class sizes have been identified as barriers to successful STEM programme implementation (Wilson, 2020). As also stated by Fullan (2007), supporting innovations at schools requires a great deal of time and financial investment. Participants should be given time to participate in these kinds of programmes within their work schedule, and there should be, for instance, resources for substitute teachers. Moreover, as also stated by the external change agents participating in this study, time should be reserved for thorough reflection after the programme has ended (Borrego & Henderson, 2014; Icel, 2018).

This study also suggests that it is important to support external change agents in finding a continuum in their work with different programmes and dividing it, for instance, between national STEM programmes and teacher education to better transfer knowhow between different entities. The challenge is that STEM programmes are often temporarily supported by external funding, after which there are no institutional structures to support the sustainability of the programme impacts (Millar et al., 2019). Resources are also needed to ensure continuity and long-term impact, such as presenting and disseminating the programme outcomes to educators not participating in the programme.

The second constraint was *collaboration challenges*, namely, challenges with involving external stakeholders, regional and national collaborations, language barriers and reaching out to less-active learning communities. Based on the experiences from the LUMA2020 programme, there is clearly a need to find ways to involve various professionals in STEM programmes to better reveal real-life relevance (Allen et al., 2019) and motivate learners to pursue future STEM studies and careers, for example, by involving science mentors as role models (Kitchen et al., 2018; van den Hurk et al., 2019). The challenges in regional and national collaborations reflect the idea that state-wide educational improvements require clear national communication and the negotiation of aspirational targets (Fullan, 2007). Future STEM programmes should endeavour to fulfil the demanding task of creating local, regional and national networks and partnerships between research institutions, educational administrators, communities, business leaders and STEM industry personnel (Basham et al., 2010).

Moreover, in countries such as Finland with more than one official language, language barriers can obstruct collaborations between learning communities representing different language groups. More attention should be paid to tackling feelings of incompetence in the language of communication in STEM collaborations at both the national and international levels (Besterman et al., 2018). Finally, while the educational outcomes of Finnish schools have traditionally been equal (OECD, 2016), there is the risk that unequal interest to participate in programmes developing STEM education can lead to between-school differences in learners’ outcomes in STEM areas. It is vital to put resources into ensuring that educational innovations developed with the external change agents and active learning communities are shared and distributed widely.

Finally, Table 3 summarises recommendations based on our findings and supported by the literature, which are likely to support the design of effective STEM programmes.

**Table 3:** Recommendations for designing effective STEM programmes

Providing versatile support: * - In-service training and workshops*, for instance, in the form of in situ example sessions with educators and their learner groups * - Materials and equipment*, consisting of both online resources and physical equipment that can be borrowed or used during field trips, as well as recommendations and instructions on how to use them * - Scaffolding* as a personalised and timely long-time support when co-designing activities with educators * - Peer support* and mutual learning experiences in the form of onsite and online meetings and forums, also between in-service and pre-service teachers * - Support from school administrations* committed to programme implementation, who liberate time for educators but also directly participate in the programme * - Extensive local, regional, national and international networks*, consisting of learning communities from all educational levels as well as STEM experts from both profit and non-profit organisations
Ensuring programme flexibility: * - Visits to learning communities* as a cost-efficient way for educators and learner groups to participate in the programme and widening programme access to those with fewer traveling possibilities * - Embedding activities in everyday work* and supporting participants directly in their work, instead of creating an additional workload * - Online communication* and online alternatives requiring less resources and, thus, widening participation * - Flexible programme objectives* that can be adapted to learning communities’ and educators’ circumstances
Maintaining long-term vision: * - Continuum from early years to adult education* with special attention to early years and creating connections between STEM initiatives in different educational levels * - Parental involvement* and involvement of other family members who positively influence young people’s future STEM career choices * - Continuum between different tasks and projects*, building up a meaningful whole of various STEM initiatives, instead of a fragmented set of activities * - Evaluating programme effectiveness* and applying the results to develop future programmes
Managing limited resources: * - Time for planning and implementation* to concretise the programme objectives with key stakeholders and adapt actions to their timetables * - Lack of financial resources* requiring scaling down the actions based on the available resources or identifying additional funding or sponsorships to support the work * - Balancing between tasks* by ensuring that they are meaningfully connected and that there is sufficient time for different duties, for instance, as a change agent and a teacher educator * - Resources for continuity and long-term impact* allocated to maintain the activities and networks after the programme finishes, for instance, to support further development, deployment, use and sharing of materials created during the programme
Tackling collaboration challenges: * - Involving external stakeholders* and motivating them to share their expertise, for instance, by means of “an expert bank” of professionals interested in raising young people’s interest in STEM studies and careers * - Regional and national collaboration*, balancing national and regional aspirations and considering possible regional differences in the programme implementation * - Language barriers* may be overcome by providing translations and supporting the use of various languages * - Reaching out towards less-active communities* to prevent or diminish between-school differences in STEM education

## Conclusion

This study provides an alternative approach for considering the effectiveness of STEM programme implementation by listening to the perspectives of external change agents. We assume that collecting feedback from STEM programme participants is already (or, at least, should be) a standard practice in impact evaluation, for example, in the form of asking the participants whether the programme objectives were fulfilled, as was done in the LUMA2020 programme. Thus, we propose the external change agent perspective supplementary for building an understanding of STEM programme implementation by interviewing programme members who were accountable for the implementation of the programme in local learning communities and communicating the local needs and challenges to the programme management. By doing so, this study demonstrated the value of external change agents’ perspectives as mediators between local programme participants and the national decision-making level in identifying enablers and constraints in effective programme implementation.

The modest number of external change agents interviewed and the lack of internal stakeholder perspectives are some of the limitations of this study. We also did not aim to quantify the qualitative data, e.g. by calculating the frequency each theme appeared in the data. Rather, we followed an interpretative approach, focusing more on meaningfulness than the frequency of themes in the data. For this reason, we did not, for example, use techniques such as counting interrater reliability. Instead, the authors discussed the interpretations of the data to reach a consensus on the themes identified as enablers and constraints. A more quantitative approach to data analysis could be chosen for future studies with a larger sample size.

The results of this study were obtained from Finland, where participation and long-term planning are part of the educational and sociocultural context (e.g. Sahlberg, 2011). For this reason, these enablers may be somewhat more natural elements in the Finnish context, while they may require more effort in contexts with different traditions. Further, the identified enablers and constraints are, of course, strongly related to the LUMA2020 programme, particularly because of the data-driven nature of our data collection and analysis method. As such, we did not want to ask the external change agents whether they agreed or not with the enablers and constraints already known in the research literature. The identified enablers and constraints resonate with existing literature and, thus, indicate that they have been encountered in other contexts as well. Hence, considering the findings from this study can help in designing effective future STEM programmes. Based on our findings, attention should be paid particularly to providing (1) various support mechanisms for educators, (2) flexibility in programme implementation, (3) a long-term vision, (4) sufficient time and economic resources and (5) collaboration and networking opportunities between various stakeholders and system levels.

The enablers and constraints identified in this study can be used in future studies, for example, by operationalising them and developing survey instruments to pursue larger sample sizes and a wider variety of participant roles. While similar enablers and constraints have been identified in the previous literature, as an added value to the existing body of literature, listening to change agents that participated in this study offers an overview of enablers and constraints based on deep practical experience with STEM programmes. In the future, more research is needed to reveal how individual enablers and constraints interact and have synergistic effects in programme implementation.

## Data Availability

The datasets used and analysed in the current study are available in the Finnish language from the corresponding author upon reasonable request.
